# Understanding the impact of gendered roles on the experiences of infertility amongst men and women in Punjab

**DOI:** 10.1186/1742-4755-10-3

**Published:** 2013-01-15

**Authors:** Zubia Mumtaz, Umber Shahid, Adrienne Levay

**Affiliations:** 1 School of Public Health, University of Alberta, 3-309 Edmonton Clinic Health Academy, 11405 – 87 Ave, Edmonton, AB T6G 1C9, Canada; 2 University of Texas School of Public Health, Houston, TX, USA

## Abstract

While infertility is a global challenge for millions of couples, low income countries have particularly high rates, of up to 30%. Infertility in these contexts is not limited to its clinical definition but is a socially constructed notion with varying definitions. In highly pronatalistic and patriarchal societies like Pakistan, women bear the brunt of the social, emotional and physical consequences of childlessness. While the often harsh consequences of childlessness for Pakistani women have been widely documented, there is a dearth of exploration into the ways in which prescribed gender roles inform the experiences of childlessness among Pakistani women and men. The aim of this study was to explore and compare how gender ideologies, values and expectations shape women’s and men’s experiences of infertility in Pakistan. Using an interpretive descriptive approach, in-depth interviews were conducted with 12 women and 8 men experiencing childlessness in Punjab, Pakistan from April to May 2008. Data analysis was thematic and inductive based on the principles of content analysis. The experience of infertility for men and women is largely determined by their prescribed gender roles. Childlessness weakened marital bonds with gendered consequences. For women, motherhood is not only a source of status and power, it is the only avenue for women to ensure their marital security. Weak marital ties did not affect men’s social identity, security or power. Women also face harsher psychosocial, social, emotional and physical consequences of childlessness than men. They experienced abuse, exclusion and stigmatization at the couple, household and societal level, while men only experienced minor taunting from friends. Women unceasingly sought invasive infertility treatments, while most men assumed there was nothing wrong with themselves. This study highlights the ways in which gender roles and norms shape the experiences associated with involuntary childlessness for men and women in Punjab, Pakistan. The insight obtained into the range of experiences can potentially contribute to deeper understanding of the social construction of infertility and childlessness in pronatalistic and patriarchal societies as well as the ways in which gender ideologies operationalise to marginalise women.

## Background

Infertility is a worldwide problem with an estimated 8-12% of couples having difficulty conceiving a child at some point in their lives, impacting upwards of 60–80 million people [1-3]. In low-income countries(LICs), despite high fertility rates, infertility rates range from 22% in South Asia to 29% in some sub-Saharan countries [2].

There is no universal definition of infertility. Generally a couple is considered clinically infertile when pregnancy has not occurred after at least twelve months of regular sexual activity without the use of contraceptives [1]. There are two clinical types of infertility. Primary infertility is caused by anatomical, genetic, endocrinological, and immunological problems leading to the inability to have a child. This form of infertility has been estimated to cause childlessness in about 5% of couples worldwide. Secondary infertility is usually due to sexually transmitted infections, poor health care practices, exposure to toxic substances, and socio-cultural practices such as endogamous marriages (marriage between relatives) and female genital mutilation [2]. Secondary infertility contributes to the majority of childlessness, particularly in LICs, and is easily preventable [2,4].

It is important to keep in mind that the definition of infertility varies between cultures and that the biomedical definition cited above may not capture variation in cultural perceptions of childlessness. A childless person or couple does not view themselves as ‘infertile’ until they define the state of childlessness as a problem [5]. In some contexts a couple with children might consider themselves infertile if they do not have the right number and right type of children [6]. In other places, infertility may be understood as having no sons, or not becoming pregnant soon after initiating sexual activity [4]. Ultimately, the notion of infertility is built upon socio-cultural perceptions, particularly perceptions regarding the value of parenthood as a social role in a given society [5].

One primary socio-cultural institution that influences notions of infertility is gender [7]. Gender refers to the socially constructed roles, behaviour, activities and attributes that a particular society appropriates for men and women. Women and men “do” gender every day, acting out these prescribed gender roles and norms [8]. Socially constructed gender ideologies shape the lives of men and women around the world [9]. Deeply rooted in men’s and women’s consciousness beginning from a birth [10], gender ideologies crucially impact sense of self and identity that cuts across social and class divisions. In most parts of the world, although not all, women’s primary identity is wife-hood and mother-hood and they are largely held responsible for procreation while men’s primary identity is that of a breadwinner and protector of their families [11].

### Infertility and gender in Pakistan

Although known for its high birth rate, infertility is a major reproductive health problem in Pakistan with estimates ranging from 15-22% [12,13]. Reproduction may be a biological phenomenon, but because of its crucial role in the formation of families, communities, and nation-states, it is also a closely regulated social and cultural behavior [9]. Pakistan is a highly pronatalistic society where rigid social values, gender norms and structures universally promote childbearing. The construction of these institutions, particularly gender, occurs at the macro-level and the micro-level.

On the macro-level, the current gender ideology in Pakistan is largely centered on a patriarchal interpretation of Islam in what is a normatively patriarchal South Asian society. Men are considered to be inherently superior beings as they are understood to possess qualities of dominance, power and authority and can be trusted with the authority over the system. Women, on the other hand, are understood to be inferior beings, primarily because of their supposed inherent emotional and physical instability. The only kind of education fit for a woman is the kind of education that prepares her to be a good wife, mother and homemaker [14]. This gender order is presented as divine and *natural*[15].

At the micro-level, gender norms are ensured through the family unit. There is a strong orientation in Pakistan towards family life and family values and what holds this unit together is that men and women each agree to fulfill their mutually exclusive yet complementary gender roles within the household [16]. The ideal Pakistani family is a joint family with a male-headed household, his wife, their grown sons with their wives and a large brood of children, all living together in peace. The desired number of children is four [17,18], with at least 2 sons [18].

In Pakistan, family formation is patrilineal [19]. Families are formed through, most often, endogamous marriages of first or second cousins [20]. A new bride takes up virilocal residence in her new marital family’s home and, while she may not find it to be an unfamiliar place, she will occupy a structurally weak position in this new home. It is here that the feminine ideal is played out through the new bride’s subordination and absolute dependence [18]. Not only is she subordinate to the men in the family but also to the other senior women of her marital family, mostly the mother-in-law [21,22].

A critical milestone in a woman’s life after marriage is motherhood. Women are expected to produce at least one son to carry on the patriline. Motherhood, preferably within the first year after marriage, is considered essential to prove a woman’s fecundity, give the family an heir and to secure her position within the marital home and within society [23,24]. Women who bear sons can ultimately, over time, ascend higher up the family hierarchy and may eventually acquire even greater decision-making power than younger men in the family [11].

Because of the immense value and important role of reproduction in this strongly patrilineal society, childlessness is not only unacceptable, it is unthinkable and viewed as a failure to adhere to cultural norms [6]. In this context, childlessness is disastrous with social consequences that have been well documented for women [25-28]. Children are believed to cement bonds that hold a husband and wife together [24] leaving childless women in unstable and precarious relationships, facing harsh social and sometimes physical consequences [1]. Women experience physical, mental and emotional abuse from husbands and their in-laws [26], taunting and verbal harassment [23], being socially excluded from important events like weddings [29] and from touching babies [1], threatened with divorce or having their husband marry a second wife, and ejection from their marital home [26]. Women have reported the psychosocial consequences to be depression, unhappiness, dejection and suicidal ideation [29].

In Pakistan, as well as a number of other locales, women are blamed for childlessness irrespective of which partner is actually clinically infertile [26,30] and regardless of well-established biomedical understandings of infertility that place men as potentially equal contributors to childlessness [31]. This gender differential has also led to a bias in the body of literature from much of the global south in that the majority of research surrounding the experience of infertility largely focuses only on women [25-27,29,32-34]. In the sparse research that has been undertaken in the global south regarding men’s experiences, men have reported stigmatization, verbal abuse, emasculation [20,35], and loss of social status [36,37]. In other studies, childless men have reported to be less motivated to work which has economic consequences for women [33,36]. Generally the literature shows that in highly patriarchal societies the contemplation of infertility in men is unusual. In India, men reported that they were aware of the possibility of themselves being the “defective” infertile partner; however they were reluctant to undertake any treatments for the fear of social disgrace [20].

While the small body of literature from Pakistan has discussed the negative implications of childlessness for women, little is known of how prescribed gender roles inform and shape Pakistani women and men’s experiences of childlessness. Given the importance of the gender order in shaping everyday lives, the aim of this study was to explore and compare the role of gender ideologies, values and expectations in shaping women’s and men’s experiences of childlessness in Pakistan.

## Methods

Using an interpretive descriptive approach, in-depth interviews were conducted with 12 women and 8 men who were childless. Of these 20 participants, only 4 were married to each other (2 couples). Participants were purposively recruited using a community-based snowballing technique. In-depth interviews were the primary and only method used because of the sensitive nature of the topic. A discussion guideline was developed and pre-tested. The questions focused primarily on women and men’s personal experiences of infertility and their coping strategies including types of health care sought. They were open ended in order to allow the participants’ experiences and interpretations to drive the conversations [38]. As data collection proceeded, our interviewing techniques became more probing as we sought to elicit a greater understanding of emerging themes.

Data were collected from April to May 2008. US and ZM conducted the interviews in Urdu or Punjabi at a time and location convenient to the participants. All participants, without exception, were interviewed individually in a private setting. Partners in particular were excluded during interviews as it was anticipated that the presence of a partner might inhibit communication. Oral informed consent was obtained from all participants. Each interview lasted between 40 and 90 minutes. The discussions were digitally recorded, transcribed verbatim and translated into English by UM.

Data analysis was thematic and inductive based on the principles of content analysis [39]. Data were coded for descriptive labels based on the key research objective of exploring the social and gendered experiences of infertility by woman and men. Codes were manually re-categorized into domains, which were then analysed to extract themes [39]. Narratives from different interviews were merged to describe typical experiences and behaviours, while the atypical were also accounted for. The codes, domains and themes were carefully considered for validity collectively by US and ZM [40].

Ethical clearance was obtained from The Ethics Committee of the Health Services Academy in Islamabad, Pakistan. The ethical case was based on the premise that all fieldwork would be conducted on a voluntary and informed basis. Names were changed in the transcripts to protect the participant’s anonymity.

## Results

### Participant characteristics

The mean age of our female and male participants was 35 and 37 years respectively. All, with the exception of two women, were currently married, women for an average of 12 years and men 9 years. Eleven out of the 12 women were childless and one had recently given birth to twins after intra-cytoplasmic sperm injection. Of the eight men, 5 were childless. Seven participants had adopted a child, although three of them had lost the adopted child, two at the time of their divorce and one due to the husband’s family taking the child back because they felt guilt for having given her away. See Tables 1 and 2 below for a more detailed description of the participants.

**Table 1:** Female participant characteristics

**Name**	**Age**	**No. of Surviving Children**	**No. of Failed Pregnancies**	**Total no. of marriages**	**Total no. of spousal marriages**	**Endogamous? (Y/N)**
Farzana	35	0	0	1	1	Y
Abida	40	0	0	1	1	Y
Sayeeda	30	0	0	1	1	Y
Mrs. Zulfiqar	35	0	0	1	1	Y
Attiya	24	0	1	1	1	Y (married to Zafar)
Zarina	39	0	0	1	3	Y (husband’s current wife), N (husband’s 1st and 3rd wife)
Nusrat	41	0	0	2	2	N (neither husbands)
Shahida	38	0	2	1	1	Y
Fatima	38	0	0	1	1	N
Rehana	38	2	0	1	1	N (married to Asif)
Saadia	37	0	3	1 (currently divorced)	2	N (neither husband’s marriages )
Bushra	29	0	0	1 (currently divorced)	4	N (none of husband’s marriages)

**Table 2:** Male participant characteristics

**Name**	**Age**	**No. of Surviving Children**	**No. of Failed Pregnancies**	**Total no. of marriages**	**Total no. of spousal marriages**	**Endogamous? (Y/N)**
Hashim	38	1	0	1	1	Y
Nasir	39	0	0	2	2	Unknown
Zafar	24	0	1	1	1	Y (married to Attiya)
Fazal Din	42	1	0	1	1	y
Sadaqat	36	0	0	1	1	Y
Asif	41	2	0	1	1	N (married to Rehana)
Ijaz	34	0	2	2	1	Y (current wife), N (first wife)
Ali	44	0	0	3	2	N (all wives)

### Infertility and weakening of marital bonds: a gendered experience

The first theme that emerged from our data is the role of children in cementing marriages. They are considered ‘*zanjeers*’ (chains) that hold a couple together. Absence of children, therefore, greatly weakened the marital bond. Of the 20 couples, 15 had weak marital ties as a result of childlessness.

‘We don’t have anything between us to stop us from leaving each other. If we had a child and my wife did not like me or I did not like her…we could not just leave each other because there would be a tie or bond between us to stop us. That is why children are important in marriage.’ (Zafar, 24 years, childless man).

However our data show that the consequences of weakened marital bonds are gendered. For women, motherhood is a source of their status and power. Although embedded in the language of *“jannat mil jati hai*” (we achieve paradise), motherhood is the only avenue for women to stake a place and ensure their marital security. In this highly patriarchal context, women are marginal members of their marital families and children, particularly sons, are the mechanism through which she cements her marital bonds and subsequent social position. Infertility, which is perceived as no children, or not having the right number and type of children (sons), therefore weakens the woman’s marital ties with her husband and his family. Such women are viewed as less committed to the marriage and prone to extra-marital affairs.

‘….they say a women’s home is made once she has a child… (aurat ka ghar tab banta hai jab uska bccha hota hai).’ (Zafar, 24 years, childless man)

The consequences of the weakening of the marital bonds can be catastrophic for women. This includes emotional and physical abuse, threat of remarriage, and the expectation that they should agree to live within a polygamous marriage. These consequences are further discussed below.

In contrast, the weakening of marital ties does not have the same social consequences for men. Men in childless marriages often withdrew from their wives mentally, emotionally and sexually. Most did not seek any treatment and usually remained uninvolved in their wives’ constant attempts to seek treatments and solutions. Some men would immerse themselves in their work, have extramarital affairs or remarry. This behaviour can be explained by the fact that weak marital ties did not affect their security, social identity and power. While their narratives described their sense of loss, fatherhood did not emerge as essential for a man’s socially engendered identity amongst our male participants as motherhood did for women.

### Abuse and harassment: stigmatization more painful than infertility itself

Our data show that, first of all, infertility is a highly stigmatizing attribute in this society, and second, that this stigma is gendered with women suffering greater stigmatization and its consequent abuse and harassment when compared to men. Goffman described stigma as ‘an attribute that is significantly discrediting’, which in the eyes of society serves to reduce the person who possesses it [41]. The stigma operates at three levels: the couple, household and societal level. We discuss each in turn.

### Abuse at the couple level

The majority of the female participants reported verbal, emotional and physical abuse from their husbands. They attributed this abuse and violence directly to their childlessness. Husbands tended to be more abusive in cases of female infertility and less so when male infertility was a factor, although there were some exceptions. A common form of emotional abuse is that the wife is expected to arrange her husband’s remarriage. Zarina was her husband’s second wife as he had divorced his first wife on the basis of childlessness. She had to arrange her husband’s third marriage, shop for the new bride’s trousseau and attend the wedding. On the wedding night Zarina was tasked with looking after the new wife’s son whom she had brought to the marriage. Zarina’s husband had never had a semen analysis.

Our data suggests men do not experience such abuse from their wives. The possibility of women’s remarriage was not even considered by either women or men. There was only one case in which the woman divorced her husband, but only after he had remarried on the grounds of childlessness.

### Abuse at the household level

More commonly reported than abuse by husband, was the abuse and stigmatization perpetuated by the marital family at the household level. The marital family invariably assumed the woman was the infertile partner, even in cases of proven male infertility. This was frequently followed by abuse that included verbal and emotional harassment and physical violence.

‘….when he told his mother (regarding his test) she did not believe that her own son is at fault….instead she blamed me…. that I practice black magic and have somehow made her son infertile. After that she would not let me give my husband tea or food because I will mix something bad in it…’ (Saadia, 37 years, childless woman)

‘….sometimes my in laws used to beat me, kick me out of the house, send me to my parents’ home….just because I could not give them a child….’ (Bushra, 29 years, childless woman)

A common situation that the women identified as a result of their childlessness was being expected to do household chores beyond normal expectations. Since they had failed to provide a child, they had become a burden to their marital families. The least they could do was compensate the marital family by doing extra household work.

‘…just because I could not give them a child….I was treated like a servant ‘ghulam’ I had no rights in the house…’(Bushra, 29 years, childless woman)

The majority of women were of the opinion that joint family residence added to the burden and sufferings of infertility, creating hurdles in development of a relationship between the couple and a lack of privacy and ability to make decisions regarding treatment options or adoption. Residence in a nuclear family was understood to allow establishment of strong bond of intimacy and closeness between husband and wife, and a reduction of interference by the in-laws due to conflicts and misunderstandings. In contrast, few men reported he had any problems in a joint family system. One man did however leave his natal family in order to keep his adopted daughter and another to escape from their sarcasm.

### Stigmatization at the societal level

All women, with the exception of one, reported systemic, societal-level stigmatization. Women described their experience in many different ways. For some it was an *‘…attitude of the people…they used to make me feel that I am somehow different…. they put me in a different category…*’ (*Rehana, 38 years, mother of twins through using ARTs).* For others, just the relentless questions about their childlessness caused pain and added to their sense of inadequacy.

Our female participants reported how they were considered ‘*manhoos*’ (bearer of bad luck). Their bad luck (*manhoosiat*) is located in the realm of the supernatural and spiritual world. They were therefore shunned and not welcomed in general, but specifically at special occasions. For example, such women were not welcomed at life affirming ceremonies such as weddings and celebrations of the birth of a new baby. Even if a childless woman does attend a wedding she is explicitly excluded from the various wedding rituals. At the birth of a new baby such women were believed to cast a ‘*saya*’ (bad spell) on the new born.

*‘I am not invited to weddings and other happy occasions…neighbours say I am* manhoos *(bringer of bad luck)…some don’t let their children near me because my ‘*saya*’ (maligned shadow) will fall over them…especially female children are kept away in case they become infertile.’ (Zarina, 39 years, childless woman)*

In contrast to women’s explicit and widespread stigmatization and abuse, men suffer minimal societal level abuse and stigmatization. Ijaz, the husband in a childless couple, said, ‘my wife used to complain what people say to her …first it was the elders in the family, but after a year, it was friends and neighbour…but till that time nobody said anything to me…everybody was targeting my wife’. Rarely were men suspected to be the infertile partner. Men themselves, and their natal families, refused to acknowledge the possibility of male infertility, even in cases of confirmed male factor infertility and they were never considered to be bearers of bad luck. Both women and men’s narratives did not provide evidence of any societal rituals that exclude childless men. At worst, men’s masculinity was questioned, but mostly by friends. Men reported hurtful comments, such as: ‘if you cannot give birth to a boy or a girl…at least try to give a shot at something in between’ (Zafar, 24 years, childless man) and ‘only real men give birth to sons. It is not something that can be done by just anybody’. (Ijaz, 34 years, childless man).

### Seeking treatment for infertility: a gendered behaviour

Deeply embedded and weaving through our data was the theme of seeking treatment for infertility, a fervent desire to do something to alleviate this problem of childlessness. This contrasts sharply with well-documented lack of demand for other reproductive health services such as pregnancy and childbirth care [42]. Moreover, our participants continued seeking infertility treatment for long periods of time, up to 20 years in some cases.

Our data also show seeking treatment for childlessness was a gendered behaviour. Always assuming women were the infertile partner, the decision to seek treatment was commonly made by women, usually their mother-in-laws, their mothers or by the women themselves. Mothers-in–law, in particular, were quick to suggest seeking care; one woman reported her mother-in-law insisted she seek infertility treatment three months after her wedding day.

More telling is which partner underwent treatment and of what type. Biomedically, a semen analysis is the first and simplest, non-invasive test for evaluation of infertility. However, men in our sample, generally refused to undertake this simple test. Men appear to view the test as a threat to their masculinity and reported being scared of a diagnosis of male infertility. It is much easier to assume the woman is the infertile partner. *‘…[my husband] was of the opinion that the defect was in me. [He would say], “*tum me nuks hai” *(the fault is yours)…there is nothing wrong in me…’ (Shahida, 38 years, childless woman)*

A few men did undergo testing, but usually only after an average of 5 years. Men also rarely undergo any treatment. Those who did seek treatment from traditional healers did so at the beckoning of friends. In contrast, women undergo numerous, expensive and invasive tests and treatments. The female partner is invariably the first to seek testing and often invasive treatment.

‘She (the healer) used to put in my vagina some bootian (herbs) …it was very painful… with burning sensation and numbness of legs….I felt nauseous all the time. In fact it was so bad, I was unable to get up for days…she continued doing this for 5 days after which we were supposed to have intercourse… that was awful…. painful…’(Abida, 40 years, childless woman)

Crucially, women continued testing and undergoing treatment even when they have been given a clean bill of health and the husbands have been identified as the infertile partner.

“….so I quietly went for hormonal therapy despite my doctor telling me not to since there was nothing wrong with me….nothing happened …then I went for follicular monitoring with clomiphene tablets….even that was normal….and I did not conceive…” (Saadia, 37 years, childless woman)

### Gender and remarriage

Divorce and remarriage as a consequence of infertility was uncommon amongst our participants. Between 20 participants and their 35 spouses past and present, there were 14 remarriages. At the same time, the majority, 13 of our 20 participants were in their first marriage irrespective of who was the infertile partner. The number of remarriages was inflated by only three men who had had three or more marriages.

Our data suggest divorce and remarriage as a consequence of infertility is a gendered phenomenon. Of the 14 remarriages, 10 were amongst men. But our empirical data also suggest the issue is more complicated than it appears. Both men and women’s narratives indicate the woman’s marital family, friends, and relatives are quick to suggest that the husband remarry if infertility is an issue, irrespective of which one is the infertile partner. Remarriage of the husband was considered a natural next step, obvious and beyond questioning.

‘Like in our family system, it is customary to remarry in case of childlessness.’ (Zafar, 24 years, childless man)

‘Though my in-laws know that their son has the problem… yet they go on and on about getting him another wife…they are willing to let me go and get another bride for him,….why are they ready to spoil another girl’s life….’ (Sayeeda, 30 years, childless woman)

Despite the threat of remarriage that hovers over women, the remarriage of men is rife with complexities. Regardless of Islam’s permission to practise polygamy, multiple marriages are a socially unacceptable practice, best exemplified by a comment made by Ijaz:

‘An uncle of mine…he had 3 daughters only….no son… so he divorced his wife and sent all the daughters with his wife…He got married again, but now God has not given him any children…not even daughters…. he is living alone with his 2nd wife…’(Ijaz, 34 years, childless man)

The men who did remarry defended their actions, reporting that either their wife left them or they were under pressure to remarry from their families and that that they had no option but to agree. Throughout their narratives, they presented themselves as passive actors whose lives were being determined by their mothers and and natal family on the one hand and their wives on the other. Our analysis of the data, however, showed that wives only left after the remarriage of their husbands. Moreover, a number of the men had repeatedly remarried despite childlessness remaining the outcome of every marriage. None of them had undergone a semen analysis. All these marriages were also exogamous (married to non-relatives).

As stated above, the majority of childless marriages remained intact. Our data suggests the key reason for this is the norm of endogamy. Endogamous marriages, the most common form of marriage in Pakistan (67% of all marriages) [43], means that a woman is not just a wife, but also the daughter of a *taya* (father’s brother) or a *khala* (mothers sister)*.* In such marriages, the marital family is under tremendous pressure for successful continuation of the relationship. A divorce may not only result in the break-up of ties between the husband and wife, but of a number of other crucial blood ties, such as between two brothers or a brother and sister.

‘My wife is my mother niece and my elder sister is married to my wife’s brother. Even they have no kids. So we have a strong family system and we do not divorce on issues of childlessness.’ (Sadaqat, 36 years, childless man)

‘My husband would divorce me today if he could…but he cannot do so because he does not have his mother’s support…she is on my side… in fact she scolds him whenever he brings up the idea of a 2nd marriage…. he is helpless. If my mother- in- law, who is also my phuphi (father’s sister) was not with me… he would have left him a long time ago.’ (Abida, 40 years, childless woman)

In the one case of an exogamous marriage that remained intact, the woman was the daughter of a rich and powerful man. The socio-cultural advantage of women benefitted them in numerous ways, including payments for high-tech infertility treatments.

I: What role did your profession (a doctor) and having family support play in your experience of infertility.

Rehana: A very positive role. I come from a very influential and rich family. If there was another girl instead of me…then maybe my in-laws could have suppressed her. They always supported me. (Woman who had given birth to twin sons after intra-cytoplasmic injections)

In contrast to the expectation that males can or should remarry for reasons of childlessness, normatively women are not expected to leave their husbands and the issue of remarriage of such women is not even a possibility that emerges for discussion.

### Adoption

When all treatment options were exhausted and remarriage was not a possibility, the possibility of adopting a child arose. By and large, our data suggest adoption is also a gendered phenomenon. Adoption is generally resisted by both women and men and their respective families. A primary reason for the strong resistance to adoption is located in the patriarchal kinship system whereby a family’s lineage is through men. If a family does decide to adopt, the preferred form of adoption is a child from the husband’s family. Moreover, generally only girls are offered for adoption, usually the third or fourth daughter. The most common types of adoptions therefore are *bhatijee* (husband’s brother’s daughter), followed by *bhanji* (husband’s sister’s daughter). Sister-to-sister adoption was rare.

Another gendered disadvantage of family adoptions is that in the event of a breakdown of the marriage, the child is returned back to the parents (commonly the family of the husband). In such adoptions, the adopted mother has no rights to the child. Furthermore, the birth parents of the adopted child can, at any time, return and take their child back from the adopted parents as in the case of Nusrat and her husband who had been given his sister’s daughter only to have to give her back after four years.

‘Last year when my daughter turned four years her real mother came…‘woh le gai meri bachi ko’ (took my daughter away)…. I was left alone….I have kept all her things and I see them daily….I miss her so much….sometime I wish I never had got this experience of having a baby and spending and sharing life with her……because she has left a big gap in my life.’ (Nusrat, 41 years, childless woman)

## Discussion

The objective of this study was to explore the ways in which prescribed gender roles, norms and values shape the experiences of childlessness including family and societal reactions, coping strategies and health seeking behaviours.

The findings from this study suggest considerable personal suffering and pain as a result of childlessness in women. These findings are in keeping with other qualitative and quantitative studies which indicate the overwhelmingly negative experiences of childless Pakistani women [6,23,26-28]. In particular, women endure long-term infertility treatments which are often painful and invasive which can result in detrimental side effects. That treatment for childlessness occurs almost entirely in the body of Pakistani women is a world-wide phenomenon [44-47].

Men’s contribution to our dataset, which is also reflected in a paucity of meaningful and relevant quotes from men, is itself a reflection of the differences in the gendered experiences of infertility. Childlessness is invariably blamed on the female partner by everybody concerned; the husband, marital family and society generally. Men in childless marriages view themselves and are seen by society as inert, usually innocent partners. Pregnancy and childbirth are considered women’s domain only. Men had little to say on the matter and their interviews lacked the depth and pain evident in women’s interviews.

Our data also show that childlessness in Pakistan greatly weakens marital bonds since children are believed to bind a couple and strengthen the marital bond, a finding previously reported [24]. Our findings however, support this body of literature as well as show that these weak marital bonds are more detrimental for women compared to men. Childless women considered themselves as *‘kamzoor’* women, women with weak social ties with their marital families [11]. Marital families suspect and question their commitment to their marriages, assuming these women are keeping their options open in terms of leaving the marriage. These weak ties rendered the childless women socially vulnerable to ostracism and abuse; in rarer cases divorce and abandonment. These findings have been reported from Bangladesh, where childless women have been accused of being more open to extramarital affairs and, as a consequence, their mobility and employment is restricted by her marital family and they are often abandoned. Very poor women are sometimes left without basic necessities like food and money [33].

However, our data includes some male perspective in addition to women’s experiences. According to our interpretation of men’s narratives, the weak marital ties as a result of childlessness do not affect men the same way as they do women because men’s social identity does not hinge on the strength of their marital ties. Mumtaz [11] shows that in a patriarchal context such as Pakistan, men’s primary ties are his blood ties: father, brothers, *chachas* (father’s brother) and *tayas* (mother’s brother). Blood ties by their nature are secure and certain. Marital ties are, for men, secondary ties.

Remarriage as a consequence of childlessness appears to be uncommon. Despite an expectation that men should remarry in the event of childlessness, in actuality it was difficult to act on. A key reason for this may be endogamy, the practice of marrying within the *birdari*[48]. In endogamous marriages, the women are not just a wife but daughter of a ‘*taya’* or a ‘*mamu*’. A breakup of an endogamous marriage can result in fracturing of key kinship ties, an unacceptable outcome in a context where social kin relations are a key dimension of individual identity [11]. The fact that endogamous marriages amongst the respondents appeared to protect women in the event of childlessness may be a new finding as it has not previously been reported in the literature.

Our third finding is the prevalence of abuse and ostracism of childless women by their husbands, marital families and society generally. This is a common finding in Pakistan and South Asia. Sami and Ali [27] found that 64% of infertile Pakistani women reported themselves to be victims of violence and this was strongly associated with infertility status. Moghadam *et al*. [32] reported physical abuse inflicted by men and in-laws on women due to their childless marriage irrespective of whose fault it is [32,34]. This abuse of women can be situated within the context of sustaining patterns of gender hegemony. As Kordvani [49] posits, men’s violence is a means to produce and reproduce masculine hegemony and sustain the gender order. When a couple is childless, while women are explicitly held responsible, it also implicitly reflects on men’s masculinity. These women, then, are held responsible for men’s emasculation, for that is what childlessness ultimately implies. Men and their families’ violence against these women is a means to reassert men’s masculinity. The large role played by the mothers-in-law and society in this violence can be explained by the notion of the intra-gender policing which must occur to ensure perpetuation of the dominant gender hegemony whereby women’s actions are policed by other women and men’s actions are policed by other men [50].

## Conclusions

It is evident then that childlessness is a highly important issue for men and women who both suffer psychosocial consequences and where women are often suffering from violent consequences as well. Provision of fertility treatments could be a starting point. But a review of the literature suggests this is a contested approach to alleviating childlessness. Some of the arguments against providing fertility treatments in LICs are that most of these countries are overpopulated was well as the fact they have limited resources and therefore providing assisted reproductive technology is a low priority in most of them [51]. In Pakistan, however, secondary infertility usually caused by a sexually transmitted infection or through previous medical procedures, accounts for the majority of infertility (18%) [13,52]. Prevention of the causes of secondary infertility could reduce the suffering of infertile couples in pronatalistic societies. Prevention is more economical, benefits more people and can benefit women, in particular, in many other unintended ways. Furthermore, programs focusing on the prevention of secondary infertility can be easily attached to existing reproductive rights and health programs [51].

According to the United Nations, reproductive health is “a state of complete mental and social well-being and not merely the absence of infirmity, in all matters relating to the reproductive system and to its functions and processes. Reproductive health therefore implies that people are able to have a satisfying and safe sex life and that they have the capability to reproduce and the freedom to decide if, when and how often to do so” [53]. This implies that all possible means of treatment and support for infertile couples, in all nations, should be exhausted in order to achieve reproductive health in a population. As noted above, the provision of treatment for infertility in LICs is contested. However, if one considers the Alma-Ata declaration that defines health as “a state of complete physical, mental and social well-being and not merely the absence of disease or infirmity” [54], there is indeed an argument for the provision of infertility treatment options, not just prevention options, for people even in developing and “overpopulated” countries. Women, in particular, are suffering consequences that are causing extremely diminished physical, mental and social well-being.

This study provides important information as it highlights the ways in which the gender order informs the experiences associated with involuntary childlessness across this heterogeneous study population in Pakistan. The insight obtained into the range of experiences can potentially contribute to the further understandings of the ways in which gender ideologies shape the everyday experiences of Pakistani women and men. Furthermore, they offer a gender lens through which to examine how these gender roles act through a phenomenon like childlessness to further marginalise women in an already highly patriarchal society.

## Abbreviation

LIC: Low-income country.

## Competing interests

The authors declare that they have no competing interests.

## Authors’ contribution

US and ZM conceptualized this research project. US undertook the data collection. ZM, US and AL analyzed the data. ZM and AL prepared the manuscript. All authors read and approved the final manuscript.
